# Daily Closeness Discrepancy and Marital Interaction Quality in Older Couples: The Mediating Effect of Loneliness

**DOI:** 10.1093/geroni/igaf122.130

**Published:** 2025-12-31

**Authors:** Yan Huang, Lynn Martire

**Affiliations:** The Pennsylvania State University, University Park, Pennsylvania, United States; The Pennsylvania State University, University Park, Pennsylvania, United States

## Abstract

Feelings of closeness are key to individual and relational well-being. However, individuals may desire different degrees of relationship closeness compared to the actual closeness they feel with their partners. Such a discrepancy in closeness may affect marital interaction quality, especially in the context of chronic pain. This study aimed to investigate associations of daily closeness discrepancy with same-day marital interaction quality and the potential mediating effect of same-day loneliness among older adults with chronic back pain (N = 147) and their partners. Participants reported daily actual and desired closeness, marital interaction quality, and loneliness for 30 days. Closeness discrepancy was defined by the absolute value of actual closeness minus desired closeness. Separate multilevel models were run for patients and partners, and the bootstrapping method was applied in multilevel mediation models. We found that closeness discrepancy on a given day was significantly related to decreased marital interaction quality that day in both patients and partners, and such daily associations were in part explained by feelings of loneliness that day. Specifically, on days when participants had higher closeness discrepancy, their levels of loneliness were also higher, and loneliness was in turn related to less positive and more negative marital interaction quality. These associations remained significant after controlling for depressive symptoms at baseline. Findings provide evidence on the daily dynamics of closeness discrepancy and marital interaction quality in the context of chronic pain and suggest loneliness as an indirect psychosocial pathway linking such daily associations.

